# Impact of HIV on Women Internationally^1^

**DOI:** 10.3201/eid1011.040624_01

**Published:** 2004-11

**Authors:** Lydia Ogden, Jessica Ogden, Promise Mthembu, Nancy Williamson

**Affiliations:** * Centers for Disease Control and Prevention, Atlanta, Georgia, USA;; † International Center for Research on Women, Washington, D.C., USA;; ‡ International Community of Women Living with HIV/AIDS, London, United Kingdom;; § Family Health International, Arlington, Virginia, USA

Women bear about half of the HIV infections worldwide. In sub-Saharan Africa, 58% of those infected are women; in Asia this figure is 30%. While the epidemic occurs in varied geographic regions, all women are biologically and socioculturally vulnerable.

Our common prevention options fail to take into account women's realities: being in, or wanting to be in, a union; wanting to have children; the imbalance of power in male/female relationships; inaccessibility of education; the threat of sexual violence; and the economic vulnerability that leads to engaging in sexual activity for survival. Female-controlled methods, including female condoms and microbicides, are essential and must take into account these realities. The prevention needs of women already infected with HIV must be addressed by supporting disclosure, fighting stigma, and being sensitive to the threat of violence and disinheritance.

The burden of care for those living with HIV/AIDS most often falls to women and girls. Recognition of the value of this work is vital, as is addressing practical issues that can help alleviate this burden of care.

## HIV-Positive Women's Perspective, Advocacy, Sexual and Reproductive Rights

Biomedical, social, and human rights factors are compelling reasons for giving particular attention to women and HIV. However, research on women and HIV/AIDS in terms of treatment, adherence, and opportunistic infections is deficient. Women lack access to treatment, and women's representation in treatment advocacy initiatives remains wanting.

In terms of sexual and reproductive health, women face barriers in accessing treatment for sexually transmitted infections and have inadequate access to prophylactic treatments such as Pap smears and sexual health screenings. Female condoms are often unobtainable, and accelerated research on woman-controlled barriers is needed. Many programs for HIV-positive women lack services to support safe conception, frequently consider women only or primarily in terms of reproduction, and can unethically deny HIV-positive women reproductive health services.

Scientific research, programs, and initiatives should focus on HIV-positive women and their interrelation with treatment, adherence, opportunistic infections, female-controlled prevention methods, and reproductive health. These findings must then be translated into ethical policy and practice.

## HIV among Young Women in Developing Countries

Youths (persons 15–24 years of age) are a major part of the HIV epidemic around the world, making up an estimated half of new HIV infections, and young women are typically infected earlier than are men. Young women have both biological and social vulnerabilities. They can be susceptible to "sugar daddy" relationships, they are vulnerable to sex trafficking or coercion, and they have less education, including HIV prevention education, than their male counterparts. Some countries have had success in reducing HIV among young women; however, many program challenges remain: lack of evaluation, limited resources, the unique vulnerabilities of youth ignored, and the lack of influence by young persons.

Fifteen million children 15 years of age and younger have lost one or both parents to AIDS, and this situation also presents challenges, including increased risk of sexual exploitation, the loss of educational opportunities as young people are forced to leave school because they lack school funds or must work to support remaining family members, and the need for HIV prevention education that addresses orphans' special needs.

Some promising youth programs have been initiated, among them curriculum-based programs, peer education, and voluntary counseling and testing; however, more resources and evaluation must be devoted to youth programs, and these programs should view youth as assets, not as problems.

